# From Environmental Financial Literacy to Sustainable Healthcare: The Mediating Role of Governance and Environmental, Social, and Governance Reporting

**DOI:** 10.3390/healthcare14203431

**Published:** 2026-10-14

**Authors:** Stefan Milojević, Snežana Knežević, Aleksandra Vujko

**Affiliations:** 1 Faculty of Medical Sciences, University of Kragujevac, Svetozara Markovića 69, 34000 Kragujevac, Serbia; 2 Faculty of Business Economics, EDUCONS University, 21208 Sremska Kamenica, Serbia; 3 Faculty of Organizational Sciences, University of Belgrade, 11000 Belgrade, Serbia; snezana.knezevic@fon.bg.ac.rs; 4 Faculty of Tourism and Hospitality Management, Singidunum University, 11000 Belgrade, Serbia

**Keywords:** Environmental Financial Literacy, ESG, governance, ESG reporting, environmental sustainability, social sustainability, sustainable healthcare

## Abstract

**Highlights:**

**What are the main findings?**

- Environmental Financial Literacy strengthens environmental and social sustainability primarily through Governance/ESG Reporting.
- Governance/ESG Reporting serves as a key mediating mechanism linking Environmental Financial Literacy to organizational sustainability.

**What are the implications of the main findings?**

- Healthcare organizations should integrate Environmental Financial Literacy development with governance practices and ESG reporting systems.
- Governance should be recognized as an enabling mechanism for environmental and social sustainability rather than merely a separate ESG pillar.

**Abstract:**

**Background/Objectives:** Healthcare organizations face challenges in integrating environmental sustainability with financial constraints, digitalization, governance, and social responsibilities. Although financial and digital financial literacy have been associated with environmental, social, and governance (ESG) performance, the organizational mechanisms underlying these relationships remain insufficiently understood. This study examines Environmental Financial Literacy (EFL) as a sustainability-relevant decision-making capability and Governance/ESG Reporting as a potential mediator linking EFL with environmental and social sustainability. **Methods:** A quantitative, cross-sectional survey included 1415 healthcare employees in Serbia. Exploratory factor analysis assessed the measurement structure, followed by reliability and validity testing. Covariance-based structural equation modeling (CB-SEM) examined the hypothesized relationships, while bias-corrected bootstrapping assessed indirect effects. **Results:** Four distinct constructs were identified. EFL was positively associated with Governance/ESG Reporting (β = 0.658, *p* < 0.001), which was positively associated with Environmental Sustainability (β = 0.561, *p* < 0.001) and Social Sustainability (β = 0.469, *p* < 0.001). Governance/ESG Reporting partially mediated both relationships, with significant indirect effects on Environmental Sustainability (β = 0.369, 95% CI [0.330, 0.415]) and Social Sustainability (β = 0.369, 95% confidence interval (CI) [0.330, 0.415]). Both indirect effects exceeded the corresponding direct effects. **Conclusions:** Governance/ESG Reporting represents a significant statistical intermediary between EFL and organizational sustainability. The findings highlight the potential importance of integrating sustainability-relevant financial competence with governance and reporting practices in healthcare organizations, while offering a framework for further investigation across different healthcare systems.

## 1. Introduction

Healthcare systems face a distinctive sustainability challenge: their mission to protect human health coexists with resource-intensive, technologically complex, financially constrained, and environmentally consequential operations [1,2,3]. Healthcare organizations consume substantial energy and materials, generate waste, and make capital-intensive decisions concerning procurement, infrastructure, medical technologies, and resource allocation [4,5,6,7]. Accounting for approximately 5% of global greenhouse-gas emissions, the health sector faces growing pressure to improve its environmental performance [8,9]. However, healthcare sustainability extends beyond environmental concerns [10,11] to encompass patient safety, employee welfare, accessibility, equality, financial viability, accountability, and stakeholder interests [12,13].

Sustainability-related decisions require healthcare organizations to evaluate environmental consequences alongside financial costs, risks, regulatory requirements, and long-term organizational implications [14,15]. Increasing digitalization further requires the effective interpretation of financial and management information [16,17,18]. Sustainable decision-making therefore depends on integrating financial reasoning, digital information, and environmental understanding [19,20]. This study conceptualizes this capability as Environmental Financial Literacy (EFL), defined as the ability to understand, evaluate, and use financial information, including digitally generated data, in decisions involving environmental costs, resource efficiency, green investments, compliance, and sustainability implications. Research increasingly connects financial and digital financial literacy with sustainability-related behavior and organizational outcomes [21,22]. Fan et al. [23] identified a positive association between digital financial literacy and corporate ESG performance among Chinese listed firms, particularly for the social dimension. International evidence similarly links financial literacy with ESG disclosure and performance [24], while individual-level studies associate digital financial literacy with sustainable investment behavior [25,26,27].

Despite these advances, existing studies primarily examine whether financial or digital financial literacy is associated with sustainability behavior, ESG disclosure, or performance [26,28,29], providing limited insight into how these capabilities translate into organizational sustainability outcomes. Financial competence alone may be insufficient without organizational structures that integrate such knowledge into strategic decisions, internal controls, risk management, reporting, and accountability [30,31]. Healthcare research identifies governance as critical to sustainability implementation [32,33], with evidence from European hospitals highlighting knowledge, management involvement, professional commitment, and technology as relevant factors [34,35]. However, this literature remains largely disconnected from research on financial and digital financial literacy [36,37]. Consequently, limited attention has been devoted to whether governance provides the organizational structures through which sustainability-relevant financial capabilities become institutionalized and associated with sustainability outcomes [38,39].

The present study examines Governance/ESG Reporting as a potential mediator linking EFL with environmental and social sustainability. Integrating financial literacy, digital financial literacy, and sustainability-oriented decision-making [25,40], EFL extends conventional financial competence by incorporating environmental costs, green investments, resource efficiency, compliance, and sustainability-related financial information into healthcare decision-making [41,42]. The framework emphasizes governance functions—including transparency, strategic decision-making, risk management, internal controls, ethical accountability, and information quality—as organizational processes through which sustainability-relevant financial competence may become associated with environmental and social outcomes.

Conventional ESG frameworks conceptualize environmental, social, and governance dimensions as parallel sustainability pillars [43,44,45], potentially obscuring their distinct organizational functions [46,47]. This study proposes that the governance dimension also performs a process-enabling role, embedding sustainability-relevant financial competence into organizational reporting, controls, accountability, risk management, and strategic decisions. Resource-based reasoning [48] and dynamic capabilities theory [49] explain how organizational capabilities become consequential through their deployment, while stakeholder theory [50] extends this reasoning to social outcomes affecting employees, patients, communities, and other stakeholders. Using data from 1415 employees across primary, secondary, and tertiary healthcare organizations in Serbia, where healthcare financing challenges, ongoing digital transformation, and the development of digital health literacy remain relevant organizational concerns [51,52,53], this study conceptualizes EFL as a sustainability-relevant decision-making capability and examines whether Governance/ESG Reporting mediates its relationships with Environmental Sustainability and Social Sustainability.

## 2. Literature Review and Hypothesis Development

### 2.1. Literature Review

Sustainability-oriented decision-making extends financial competence beyond conventional budgeting, investment, and risk assessment [54] to include environmental costs, resource efficiency, regulatory requirements, sustainability risks, and long-term investment implications [55,56]. Digital technologies increasingly support these decisions through monitoring, analysis, and reporting [57], integrating financial, digital, and sustainability-related knowledge [25,28]. Empirical evidence links financial and digital financial literacy with sustainable investment behavior and corporate ESG performance [27,58], while international studies identify positive associations with ESG disclosure and performance [36,40]. Within healthcare organizations, EFL informs decisions concerning energy consumption, medical waste, procurement, infrastructure, and environmental compliance by integrating financial, digital, and environmental considerations [41,59,60]. Although sustainability is increasingly integrated into healthcare management and control systems [61,62], the organizational mechanisms connecting EFL with sustainability outcomes remain insufficiently understood.

The resource-based view suggests that organizational capabilities contribute to outcomes when deployed through value-creating strategies [48], while dynamic capabilities theory emphasizes their integration, coordination, and adaptation [49]. Accordingly, EFL represents a sustainability-relevant decision-making capability integrating financial, environmental, and digital information [63], whose organizational relevance depends on its incorporation into governance systems. Governance provides structures for oversight, responsibility allocation, risk management, internal controls, strategic decisions, transparency, and accountability [64,65], consistent with contemporary governance research emphasizing administration, disclosure, control, and organizational decision-making [66,67]. EFL may strengthen governance by integrating environmental costs, investments, sustainability risks, and digitally generated financial information into strategic decisions, monitoring, controls, and ESG reporting [68]. Empirical evidence linking digital financial literacy with ESG performance and governance outcomes [69], and financial literacy with ESG disclosure [70], supports this proposed relationship.

Governance institutionalizes sustainability priorities through recurring organizational decisions [39], including those concerning energy consumption, resource efficiency, waste management, procurement, environmental risks, and investments [71,72]. These activities require accountability, internal controls, reliable information, and the integration of environmental considerations into decision-making [73]. Existing research associates sustainability governance with ESG performance [74], corporate governance with sustainability outcomes [75], and governance configurations with environmental performance through strategic and organizational processes [76]. In healthcare, Governance/ESG Reporting may support environmental sustainability through transparency, accountability, risk management, and monitoring, particularly when environmental initiatives involve substantial resources and operational consequences [77,78].

Stakeholder theory emphasizes incorporating diverse stakeholder interests into organizational decision-making [50], a particularly important responsibility in healthcare, where patients, employees, communities, regulators, suppliers, and funding bodies have differing expectations [79,80]. Social sustainability encompasses employee welfare, professional development, equality, patient safety, rights, accessibility, and stakeholder relationships [73], requiring organizational mechanisms that systematically integrate these responsibilities into decisions [73,81]. Governance supports stakeholder integration through transparency, accountability, reliable information, risk management, and strategic oversight [82]. Evidence associates these mechanisms with social and environmental performance [83], suggesting that Governance/ESG Reporting may help healthcare organizations monitor stakeholder responsibilities and incorporate social considerations into decision-making [84,85]. Together, these arguments suggest that EFL may support environmental and social sustainability through Governance/ESG Reporting, which enables organizational capabilities to be deployed and embedded in recurring practices [48,49].

EFL may remain confined to individual decision-making unless integrated into organizational systems [86]. Governance/ESG Reporting provides a potential mechanism through which this capability becomes embedded in strategic decisions, reporting, controls, risk management, and accountability [87], thereby connecting EFL with organizational sustainability outcomes. For environmental sustainability, this mechanism may institutionalize financial evaluations of environmental costs, investments, risks, and resource implications [88,89,90]. For social sustainability, governance may link financially informed decisions with accountability toward employees, patients, and other stakeholders [91,92].

### 2.2. Hypothesis Development

Based on the preceding theoretical arguments, five hypotheses are proposed:

**H1.**  *Environmental Financial Literacy is positively associated with Governance/ESG Reporting.*

**H2.**  *Governance/ESG Reporting is positively associated with Environmental Sustainability.*

**H3.**  *Governance/ESG Reporting is positively associated with Social Sustainability.*

**H4.**  *Governance/ESG Reporting mediates the relationship between Environmental Financial Literacy and Environmental Sustainability.*

**H5.**  *Governance/ESG Reporting mediates the relationship between Environmental Financial Literacy and Social Sustainability.*

### 2.3. Conceptual Model

**Figure 1:**
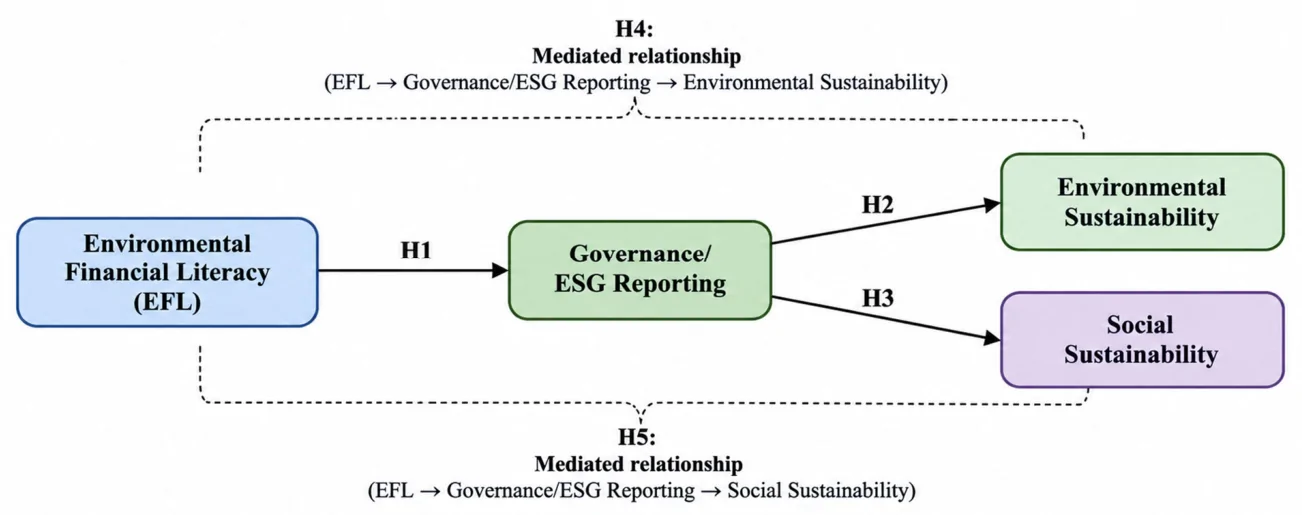
Conceptual model and hypothesized relationships.

The conceptual model, presented in Figure 1, positions Governance/ESG Reporting as a mediator between EFL and Environmental and Social Sustainability, while retaining direct relationships between EFL and both outcomes. It integrates resource-based reasoning, which conceptualizes EFL as a valuable organizational capability [48]; dynamic capabilities theory, which explains its organizational deployment [49]; and stakeholder theory, which extends its potential implications to employees, patients, communities, and other stakeholders [50].

## 3. Materials and Methods

### 3.1. Data Collection and Sample

The study employed a quantitative, observational, cross-sectional survey design with an explanatory-correlational approach to examine the relationships among Environmental Financial Literacy, Governance/ESG Reporting, Environmental Sustainability, and Social Sustainability in healthcare organizations. The design also enabled the assessment of the proposed statistical mediating role of Governance/ESG Reporting without implying temporal or causal relationships. Following ethical approval by the Ethics Committee of the Faculty of Medical Sciences, University of Kragujevac (Approval No. 09-9518/6, 31 August 2026), data were collected from 1 to 10 September 2026 using a structured questionnaire administered to employees of healthcare organizations.

A non-probability, institutionally facilitated sampling strategy was employed to obtain respondents from different healthcare professions, organizational levels, and healthcare settings. Participating healthcare organizations served as access points to eligible employees but were not involved in completing or evaluating individual responses. To enhance institutional and geographical coverage, recruitment was conducted concurrently across multiple healthcare organizations located in different parts of Serbia. Institutional diversity was addressed by including employees from primary, secondary, and tertiary healthcare organizations, while geographical coverage was pursued through recruitment across different locations rather than concentrating data collection within a single healthcare institution or locality. Access to eligible participants was coordinated through designated institutional representatives, and a mixed-mode approach combining on-site recruitment and electronic questionnaire distribution was employed to facilitate participation across healthcare settings. Given the non-probability sampling strategy, this approach was intended to increase sample heterogeneity rather than establish geographical representativeness.

During institutional visits, eligible healthcare employees were approached directly and invited to participate. In parallel, designated institutional representatives facilitated electronic distribution of the same questionnaire by forwarding a survey link to eligible employees within their organizations. A total of 1438 questionnaires were collected between 1 and 10 September 2026. Following data screening, 23 questionnaires were excluded because they were incomplete or improperly completed, resulting in a final analytical sample of 1415 valid responses (98.4% of all collected questionnaires).

### 3.2. Questionnaire Development and Content Validation

The study employed a structured questionnaire developed specifically for the healthcare organizational context. The initial measurement instrument comprised 51 items addressing financial and digital financial literacy, sustainability-oriented financial and organizational decision-making, environmental and social organizational practices, governance and ESG reporting, and the use of digital technologies in healthcare organizations. All measurement items were assessed using a five-point Likert-type scale ranging from 1 (“strongly disagree”) to 5 (“strongly agree”). The complete initial item pool is presented in Appendix A.

The four constructs were operationally defined according to their respective measurement domains. Environmental Financial Literacy (EFL) refers to respondents’ reported capability to evaluate environmental costs, green and sustainable investments, resource efficiency, environmental reporting, and regulatory compliance using sustainability-relevant financial information [19,23,24,54,59]. Environmental Sustainability captures reported organizational practices concerning energy efficiency, resource monitoring and reduction, environmental impact, waste management, green procurement, and environmental training [1,6,7,60,61,71]. Social Sustainability reflects reported organizational practices related to employee welfare, professional development, equality, patient safety, patient rights, and stakeholder responsibilities [50,73,78,81]. Governance/ESG Reporting captures reported organizational arrangements involving financial transparency, strategic decision-making, risk management, internal controls, ethical accountability, and information quality [15,64,65,66,67,68,78]. The operational meaning of each questionnaire item, together with its supporting conceptual references, is provided in Appendix A. These references informed the conceptual development of the instrument and do not indicate that the items were adopted verbatim from previously validated scales.

The questionnaire was literature-informed and contextually developed rather than derived from the direct adoption of a single pre-existing measurement scale. Item formulation was guided by several interconnected streams of literature. First, research on financial and digital financial literacy provided the conceptual basis for items capturing the ability to interpret financial information, evaluate financial consequences, use digitally supported financial information, and incorporate financial considerations into organizational decision-making. Recent studies have linked financial and digital financial literacy with financial decision-making, sustainable financial capability, sustainable investment choices, and ESG-related outcomes [19,23,25,54]. In particular, evidence that digital financial literacy can contribute to corporate ESG performance and that financial literacy can enhance ESG disclosure provided a basis for extending conventional financial-literacy concepts toward sustainability-related organizational decision-making [23,24].

Second, the development of items addressing governance, ESG reporting, accountability, and sustainability-oriented organizational practices was informed by research positioning governance as an organizational mechanism through which financial, digital, and managerial capabilities can be translated into sustainable organizational outcomes [37,40,68,76]. This literature emphasizes the connections among financial literacy, governance mechanisms, ESG disclosure, accountability, and environmental performance, providing the conceptual basis for treating governance and reporting as organizational processes rather than merely formal compliance activities.

Third, the healthcare-specific formulation of the items was informed by research addressing environmental sustainability, governance, accountability, resource management, and sustainability reporting in healthcare organizations [1,15,32,61]. This literature highlights the importance of organizational governance, environmental management, management controls, and reporting mechanisms for embedding sustainability into healthcare operations and decision-making. This literature was used to contextualize broader financial-literacy and ESG concepts within the operational realities of healthcare organizations rather than to reproduce existing healthcare scales verbatim.

Prior to data collection, the initial 51-item questionnaire underwent expert content validation. Three independent experts, all holding doctoral degrees and possessing academic and research expertise relevant to healthcare management, sustainability/ESG, financial literacy, and quantitative research, independently assessed the instrument. Each item was evaluated in terms of relevance, clarity, comprehensibility, conceptual consistency, and appropriateness for the healthcare organizational context. The experts additionally considered whether the overall item pool provided adequate coverage of the conceptual domains addressed by the study. Based on their assessment, item wording and contextual applicability were reviewed and refined before administration. This procedure was undertaken to establish the content and face validity of the initial instrument.

Importantly, no empirical factor structure was imposed a priori. All 51 measurement items were administered in the main survey, and their latent dimensional structure was subsequently investigated using exploratory factor analysis (EFA). The final composition of the measurement instrument was therefore empirically derived. Following examination of factor loadings, cross-loadings, and the theoretical interpretability of the emerging factor solution, 41 of the original 51 items were retained and 10 were removed. Items were excluded when they exhibited insufficient factor loadings, problematic cross-loadings, or did not contribute adequately to a theoretically coherent and interpretable factor. Item retention was consequently based on the combined consideration of statistical evidence and conceptual interpretability, rather than on the mechanical application of a single loading threshold. The complete original item pool, together with the retention status and final factor allocation of each item, is reported in Appendix A.

### 3.3. Statistical Analysis

The dimensional structure of the initial item pool was examined using exploratory factor analysis (EFA) with maximum likelihood extraction. Sampling adequacy and factorability were evaluated using the Kaiser–Meyer–Olkin (KMO) measure and Bartlett’s test of sphericity. Because the underlying dimensions were theoretically expected to be correlated, Promax oblique rotation with Kaiser normalization was applied. Factor retention was informed by eigenvalues, factor-loading patterns, conceptual interpretability, and the stability of the resulting structure. Items were evaluated based on their primary loadings, cross-loadings, and theoretical coherence. Statistical analyses were conducted using IBM SPSS Statistics and IBM SPSS AMOS (version 28.00).

Following EFA, questionnaire reliability was evaluated at the construct level using Cronbach’s alpha (α), corrected item–total correlations, and composite reliability (CR). Cronbach’s alpha was used to assess the internal consistency of the items within each identified construct, while corrected item–total correlations were examined to evaluate the contribution of individual items to their respective scales. Composite reliability was additionally calculated to assess the internal consistency of the latent constructs while accounting for their standardized factor loadings. Cronbach’s alpha and CR values of 0.70 or higher were considered indicative of acceptable internal consistency. Construct validity was assessed separately through average variance extracted (AVE) for convergent validity and the Fornell–Larcker criterion and heterotrait–monotrait ratio (HTMT) for discriminant validity. Potential common method variance arising from the use of a single self-report questionnaire was assessed using Harman’s single-factor test.

The hypothesized relationships among the latent constructs were subsequently tested using covariance-based structural equation modeling (CB-SEM) with maximum likelihood estimation. Model fit was evaluated using multiple absolute, incremental, and parsimony-adjusted fit indices, including χ^2^/df, RMR, GFI, AGFI, NFI, IFI, TLI, CFI, RMSEA, PNFI, and PCFI. Direct effects were evaluated using standardized path coefficients (β), standard errors, critical ratios, and associated *p*-values. The mediating role of Governance/ESG Reporting was examined using bias-corrected bootstrapping with 5000 bootstrap resamples. The statistical significance of indirect effects was determined using 95% bias-corrected bootstrap confidence intervals. An indirect effect was considered statistically significant when its confidence interval did not include zero. Mediation was classified as partial when both the indirect effect and the corresponding direct effect remained statistically significant, and as full when the indirect effect was significant while the corresponding direct effect was no longer statistically significant. Finally, R^2^ values were examined to assess the proportion of variance explained in each endogenous construct.

## 4. Results

### 4.1. Sample Characteristics

The sociodemographic and professional characteristics of the respondents are presented in Table 1. The sample (N = 1415) was predominantly male (62.4%) and consisted largely of experienced healthcare employees, with 70.4% aged over 40 years and 72.3% reporting more than ten years of professional experience. Participants represented all three levels of healthcare delivery, with the largest proportion working in secondary healthcare (43.4%). Public healthcare organizations predominated (77.7%), while both managerial (22.3%) and non-managerial (77.7%) employees were represented. The sample also exhibited variation in professional roles, educational attainment, and ESG implementation status, with 72.9% reporting that ESG principles were fully or partially implemented or that implementation was underway. Overall, these characteristics provide a heterogeneous professional and organizational context for examining the proposed relationships, although the predominance of public-sector respondents limits the generalizability of the findings to privately owned healthcare organizations.

### 4.2. Measurement Model

The Kaiser–Meyer–Olkin (KMO) measure of sampling adequacy was 0.977, indicating excellent sampling adequacy for factor analysis. Bartlett’s test of sphericity was statistically significant, χ^2^(1275) = 29,913.641, *p* < 0.001, confirming the suitability of the correlation matrix for factor extraction. Four factors had initial eigenvalues greater than 1 and jointly accounted for 46.75% of the total variance prior to rotation. The first four factors accounted for 27.57%, 11.17%, 5.14%, and 2.86% of the variance, respectively. Items with factor loadings below 0.40, problematic cross-loadings, or inadequate conceptual contribution to a clearly interpretable factor were removed from the measurement structure. Following this procedure, 41 of the original 51 items were retained. Maximum likelihood extraction yielded an interpretable four-factor solution comprising Environmental Sustainability, Social Sustainability, Environmental Financial Literacy, and Governance/ESG Reporting. As shown in Table 2, the retained items loaded substantially on their respective factors, supporting the identified four-factor structure.

Reliability analysis indicated good to high internal consistency across all four factors. Cronbach’s α was 0.919 for Environmental Sustainability, 0.923 for Social Sustainability, 0.836 for Environmental Financial Literacy, and 0.830 for Governance/ESG Reporting. Corrected item–total correlations ranged from 0.546 to 0.733, 0.544 to 0.740, 0.550 to 0.661, and 0.516 to 0.674, respectively, supporting the reliability of all four measurement scales (Table 2).

Harman’s single-factor test was conducted to assess potential common method variance. The first unrotated component accounted for 27.57% of the total variance, below the conventional 50% threshold. This result suggests that no single factor dominated the variance and provides no indication of substantial common method variance based on this diagnostic.

Construct reliability and convergent validity were assessed using Cronbach’s alpha (α), Dijkstra–Henseler’s rho_A (ρA), composite reliability (ρC), and average variance extracted (AVE). As presented in Table 3, Cronbach’s alpha values ranged from 0.830 to 0.923, rho_A values from 0.839 to 0.926, and composite reliability values from 0.832 to 0.923, indicating satisfactory reliability across all four constructs. However, AVE values ranged from 0.447 to 0.461 and remained below the conventional 0.50 threshold, suggesting that convergent validity was not fully established according to this criterion. The rho_A estimates were obtained as a supplementary PLS Mode A reliability analysis, while the primary structural model was estimated using CB-SEM.

Discriminant validity was assessed using the Fornell–Larcker criterion and the heterotrait–monotrait ratio (HTMT). As presented in Table 4, the square roots of AVE ranged from 0.669 to 0.679 and exceeded the corresponding inter-construct correlations, which ranged from 0.340 to 0.660. The HTMT values ranged from 0.339 to 0.659, remaining below the conservative threshold of 0.85. Accordingly, both criteria indicated adequate discriminant validity among the four latent constructs.

The structural model demonstrated a good fit to the data. The chi-square statistic was non-significant, χ^2^(774) = 827.924, *p* = 0.087, with χ^2^/df = 1.070. The absolute fit indices were RMR = 0.029, GFI = 0.973, and AGFI = 0.970, while the incremental fit indices were NFI = 0.967, IFI = 0.998, TLI = 0.998, and CFI = 0.998. RMSEA was 0.007 (90% CI [0.000, 0.011], PCLOSE = 1.000), and the parsimony-adjusted indices were PNFI = 0.913 and PCFI = 0.942. Taken together, these indices indicated an adequate fit of the structural model to the observed data.

**Figure 2:**
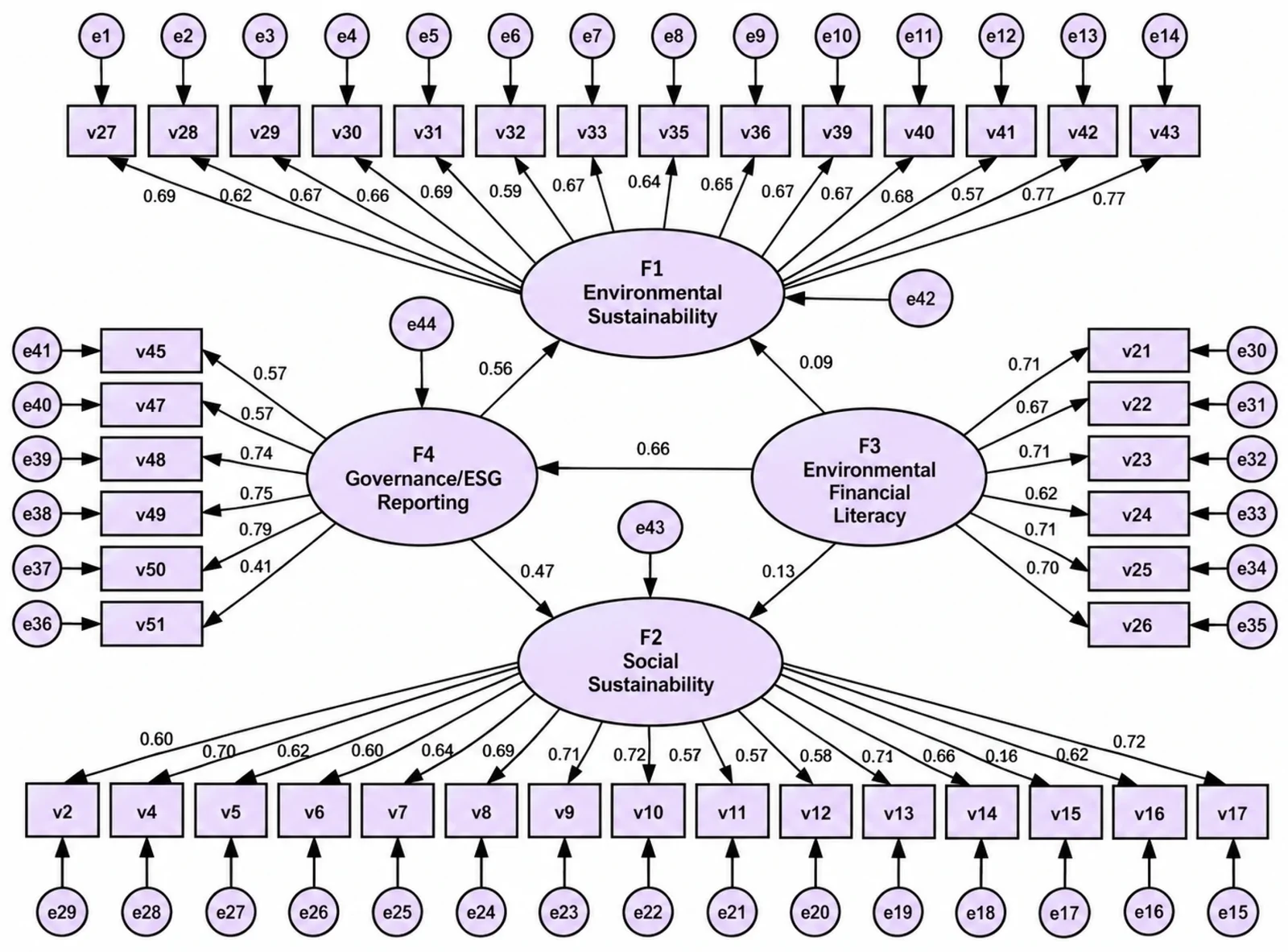
Structural Model of the Relationships among Environmental Financial Literacy, Governance/ESG Reporting, and Sustainability Outcomes.

Figure 2 presents the estimated structural model linking Environmental Financial Literacy, Governance/ESG Reporting, Environmental Sustainability, and Social Sustainability. The structural model results, including standardized path coefficients, standard errors, critical ratios, statistical significance, and explained variance, are presented in Table 5. All three hypothesized direct relationships were statistically significant and positive. Environmental Financial Literacy was positively associated with Governance/ESG Reporting (β = 0.658, *p* < 0.001), supporting H1. Governance/ESG Reporting was positively associated with Environmental Sustainability (β = 0.561, *p* < 0.001) and Social Sustainability (β = 0.469, *p* < 0.001), supporting H2 and H3, respectively. The model explained 43.3% of the variance in Governance/ESG Reporting, 39.1% in Environmental Sustainability, and 31.5% in Social Sustainability, indicating meaningful explanatory capacity across the three endogenous constructs.

The mediation analysis provided support for both mediation hypotheses. As shown in Table 6, Governance/ESG Reporting significantly mediated the relationship between Environmental Financial Literacy (EFL) and Environmental Sustainability. The standardized indirect effect was β = 0.369, with a 95% bias-corrected bootstrap confidence interval of [0.330, 0.415]. Because the confidence interval did not include zero, the indirect effect was statistically significant. The direct effect of EFL on Environmental Sustainability also remained statistically significant (β = 0.092, *p* = 0.014), indicating partial mediation and supporting H4. Similarly, Governance/ESG Reporting significantly mediated the relationship between EFL and Social Sustainability. The standardized indirect effect was β = 0.309, with a 95% bias-corrected bootstrap confidence interval of [0.266, 0.353], which also excluded zero. The corresponding direct effect remained statistically significant (β = 0.127, *p* = 0.001), indicating partial mediation and supporting H5. In both relationships, the standardized indirect effects were substantially larger than the corresponding direct effects.

Overall, the structural model provided consistent support for the proposed relationships. The findings indicate that Environmental Financial Literacy is associated with sustainability outcomes primarily through Governance/ESG Reporting, highlighting governance as the central mechanism linking financial capability with broader organizational sustainability.

## 5. Discussion

### 5.1. From Environmental Financial Literacy to Sustainability: Governance as the Missing Link

The findings reveal a strong positive relationship between Environmental Financial Literacy (EFL) and Governance/ESG Reporting (β = 0.658, *p* < 0.001), supporting H1 and aligning with previous evidence linking financial and digital financial literacy to ESG-related organizational outcomes [23,24,69,70]. By integrating financial reasoning, digital information use, and environmental considerations, EFL extends this relationship to sustainability-oriented decision-making in healthcare. Consistent with the resource-based view [48] and dynamic capabilities perspective [49], the findings suggest that sustainability-relevant financial competence is associated with governance arrangements supporting transparency, accountability, internal controls, and ESG reporting, without implying that such competence automatically generates sustainable organizational outcomes. The observed relationship further distinguishes decision-making competence from its organizational application, suggesting that governance and reporting arrangements may facilitate the integration of sustainability-related financial knowledge into organizational practices.

### 5.2. Governance/ESG Reporting as a Transmission Mechanism

Governance/ESG Reporting significantly mediated the relationships between EFL and Environmental Sustainability (β = 0.369, 95% BC CI [0.330, 0.415]; H4) and Social Sustainability (β = 0.309, 95% BC CI [0.266, 0.353]; H5). Both indirect effects exceeded the corresponding statistically significant direct effects, indicating partial mediation. Extending research linking financial literacy with ESG disclosure and performance [23,24,69,70], these findings identify governance and reporting as a potential organizational intermediary, consistent with dynamic capabilities theory [49]. However, the cross-sectional design does not establish causal or temporal mediation. The partial mediation findings indicate that EFL remains associated with both sustainability dimensions beyond the relationships statistically accounted for by Governance/ESG Reporting. These remaining direct associations may reflect the relevance of financial competence to resource allocation, investment evaluation, environmental costs, and organizational responsibilities. Governance and reporting arrangements may additionally facilitate the integration of these considerations into recurring decisions through transparency, risk management, internal controls, and accountability, representing an important but non-exclusive intermediary.

### 5.3. Why Governance Matters for Both Environmental and Social Sustainability

Governance/ESG Reporting was positively associated with Environmental Sustainability (β = 0.561, *p* < 0.001; H2) and Social Sustainability (β = 0.469, *p* < 0.001; H3). The environmental relationship aligns with previous governance research [74,75,76], while the social relationship is consistent with stakeholder theory [50] and evidence linking governance to stakeholder-oriented outcomes [83]. In healthcare, these associations highlight the relevance of governance to both environmental management and responsibilities toward patients, employees, and communities. Although the environmental coefficient was numerically larger, the statistical significance of this difference was not established. For environmental sustainability, EFL may support the evaluation of environmental costs, green investments, and resource efficiency, while governance and reporting arrangements may facilitate the integration of such considerations into procurement, resource allocation, risk management, and monitoring. The relationship observed for H2 is therefore consistent with the importance of both sustainability-relevant competence and organizational processes supporting its application.

For Social Sustainability, governance may facilitate the consideration of employee welfare, patient safety, equality, accessibility, and community responsibilities within organizational decisions. Consistent with stakeholder theory [50], financially informed decisions concerning investments, procurement, technologies, and resource allocation can be evaluated alongside stakeholder interests. The relationship observed for H3 therefore suggests that Governance/ESG Reporting is relevant to socially responsible organizational practices alongside environmental management.

### 5.4. From Decision-Making Competence to Organizational Capability

The findings distinguish individual decision-making competence from the organizational capacity to apply sustainability-relevant knowledge. EFL reflects the ability to evaluate environmental and financial information, whereas Governance/ESG Reporting represents arrangements supporting its organizational application. The strong EFL–Governance/ESG Reporting association (β = 0.658) and significant indirect relationships with both sustainability dimensions are consistent with dynamic capabilities theory [49], which emphasizes the coordination and deployment of organizational competences. Although the cross-sectional design cannot establish capability development over time, the findings highlight the potential importance of governance arrangements in connecting individual competence with organizational sustainability.

### 5.5. Repositioning the “G” in ESG

The findings suggest that governance may perform a process-enabling function alongside its conventional role as an ESG dimension. Its positive relationships with Environmental Sustainability (β = 0.561) and Social Sustainability (β = 0.469), together with the significant indirect relationships involving EFL, support examining Governance/ESG Reporting as an organizational intermediary. Consistent with resource-based reasoning [48] and dynamic capabilities theory [49], governance may facilitate the integration of sustainability-relevant competence into strategic decisions, accountability, reporting, and operational practices. These findings do not establish a universal causal hierarchy among ESG dimensions, but indicate that the intermediary role of Governance/ESG Reporting is consistent with the relationships examined in this cross-sectional model. Integrating dynamic capabilities theory and stakeholder theory [50], the results suggest that governance may connect sustainability-relevant competences with environmental priorities and stakeholder responsibilities. Its theoretical significance therefore lies in its potential dual role as both an ESG dimension and an organizational process associated with environmental and social sustainability.

### 5.6. Environmental Financial Literacy as a Boundary-Spanning Strategic Capability

The findings support conceptualizing EFL as a boundary-spanning capability integrating financial reasoning, digital information, and environmental considerations. Its positive association with Governance/ESG Reporting (β = 0.658, *p* < 0.001), alongside significant direct and indirect associations with both sustainability dimensions, suggests organizational relevance beyond conventional financial literacy. Consistent with dynamic capabilities theory [49], EFL may facilitate the integration of environmental costs, sustainable investments, resource efficiency, and digitally supported financial information across organizational decisions. The capability-based framework may also inform research on other specialized competences, including digital, data, artificial intelligence, climate, and sustainability literacy. Governance and reporting arrangements may represent organizational conditions associated with their application [48,49]. Although these domains were not examined directly, future research could investigate whether similar relationships emerge across different organizational and sectoral contexts.

### 5.7. Why the Healthcare Context Matters

The healthcare context is particularly relevant because financial constraints, technological transformation, environmental responsibilities, and stakeholder expectations frequently intersect [93,94]. Among Serbian healthcare employees, EFL was strongly associated with Governance/ESG Reporting (β = 0.658), which was positively associated with Environmental Sustainability (β = 0.561) and Social Sustainability (β = 0.469). These relationships suggest that governance and reporting arrangements may help healthcare organizations integrate financial, environmental, and social considerations into decisions concerning energy efficiency, technologies, waste management, procurement, and infrastructure. The significant indirect relationships suggest potential complementarity between EFL and Governance/ESG Reporting in healthcare sustainability management. EFL may support the evaluation of environmental costs, investments, and resource trade-offs, while governance arrangements may facilitate the integration of these considerations into monitoring, accountability, and strategic decisions. Although causal benefits cannot be established, the findings support considering financial competence and governance arrangements together when designing sustainability-oriented management practices.

### 5.8. Theoretical Contributions

The study makes three theoretical contributions. First, it conceptualizes EFL as an integrative capability combining financial reasoning, digital information, and environmental considerations. Its positive association with Governance/ESG Reporting (β = 0.658) supports the organizational relevance of this conceptualization, although its broader applicability requires further investigation. Second, the findings extend resource-based and dynamic capabilities reasoning [48,49] by identifying Governance/ESG Reporting as a significant statistical mediator between EFL and both sustainability dimensions. The partial mediation effects are consistent with a conceptual distinction between capability possession and its potential organizational application, without establishing a causal sequence. Third, the findings suggest that Governance/ESG Reporting may perform a process-enabling function beyond its conventional role as an ESG dimension. Its positive associations with environmental and social sustainability, together with stakeholder theory [50], highlight the relevance of governance to responsibilities toward employees, patients, communities, and other stakeholders. Together, these contributions advance a capability-based interpretation of healthcare sustainability.

### 5.9. Practical and International Implications

The findings suggest that healthcare administrators and policymakers should consider developing sustainability-related financial competence alongside governance arrangements supporting transparency, accountability, and reporting. Professional development programs could integrate environmental cost assessment, digital financial information management, sustainable investment evaluation, and ESG-related decision-making, particularly for employees involved in procurement, resource allocation, infrastructure planning, and environmental management. The positive relationships between Governance/ESG Reporting and both sustainability dimensions suggest that healthcare sustainability initiatives should integrate environmental and stakeholder considerations into planning, monitoring, and accountability. Accordingly, competence development and governance improvement may be approached as complementary organizational priorities.

The findings may also be relevant beyond Serbia, as healthcare systems internationally face financial constraints, digital transformation, environmental responsibilities, and increasing accountability expectations. In settings with developing ESG frameworks, integrating EFL with governance and reporting practices may offer a direction for organizational development. Healthcare systems with established sustainability frameworks may instead evaluate how effectively existing governance arrangements integrate financial and environmental knowledge into decisions. The international applicability of these findings remains dependent on institutional conditions, including healthcare financing, regulation, organizational autonomy, digital infrastructure, and ESG reporting requirements. The proposed framework should therefore not be interpreted as universally applicable, and future cross-national research is needed to assess its consistency across healthcare systems.

## 6. Limitations and Future Research

The primary limitation of this study concerns its national context. The data were collected from healthcare organizations in Serbia; consequently, the findings should be validated across different national healthcare systems, which may differ in regulatory arrangements, financing structures, digital maturity, and ESG practices. Future studies could employ longitudinal designs and incorporate objective sustainability indicators. Further research should also examine whether Governance/ESG Reporting operates similarly in other organizational and sectoral contexts and explore additional mechanisms through which Environmental Financial Literacy may influence sustainability. Finally, organizational position represents a particularly relevant extension: comparing managers and non-managers could reveal whether the translation of Environmental Financial Literacy into governance practices depends on decision-making authority and organizational responsibility.

## 7. Conclusions

This study provides evidence that the sustainability implications of Environmental Financial Literacy in healthcare extend beyond the possession of financial and digital knowledge. By examining environmental and social sustainability within a single structural framework, the study shows that the organizational conditions through which such knowledge is mobilized are central to understanding its broader consequences. The findings have a clear implication for healthcare management. Strengthening Environmental Financial Literacy should not be approached as an isolated training objective. Healthcare organizations seeking to improve sustainability should simultaneously develop the organizational conditions that allow financially informed environmental decisions to be incorporated into formal management processes. This requires closer integration between capability development, ESG information systems, strategic planning, risk management, internal controls, and sustainability-related decision-making. Such integration is particularly relevant in healthcare, where environmental objectives must be pursued alongside financial constraints and responsibilities toward patients, employees, and other stakeholders.

For healthcare decision-makers, the results also caution against relying on either competence-building or formal ESG systems in isolation. Training can increase the capacity to recognize environmental costs, risks, and investment opportunities, but organizations must also ensure that this capacity can influence actual decisions. Similarly, expanding ESG reporting requirements without strengthening the ability of decision-makers to interpret and use sustainability-related financial information may increase formal compliance without necessarily producing corresponding organizational change. Sustainability strategies should therefore align human capabilities with the systems through which decisions are made, evaluated, and monitored. The Serbian healthcare setting provides an important empirical basis for these conclusions while also defining their immediate scope. For policymakers and healthcare administrators, this points to the value of treating sustainability competence, digitalization, and governance development as interconnected elements of institutional modernization rather than as separate policy agendas.

Ultimately, the study redirects attention from a relatively simple question—whether greater financial literacy is associated with sustainability—to a more consequential organizational question: what must healthcare organizations put in place for sustainability-relevant financial knowledge to make a difference in practice? Developing knowledgeable decision-makers is only part of that challenge; equally important is creating governance conditions through which sustainability-relevant knowledge can be translated into consistent and responsible organizational action.

## Figures and Tables

**Table 1:** Sample Characteristics of Respondents (N = 1415).

Characteristic	Category	n	%
Gender	Male	883	62.4
	Female	532	37.6
Age	Up to 30 years	243	17.2
	31–40 years	174	12.3
	41–50 years	314	22.2
	51–60 years	558	39.4
	Over 60 years	126	8.8
Educational attainment	Secondary education	543	38.4
	Higher vocational education	249	17.6
	Undergraduate academic studies	277	19.6
	Master’s/Magister/equivalent	202	14.3
	Doctoral studies	81	5.7
	Integrated studies in medicine/dentistry/pharmacy	59	4.2
Healthcare experience	Less than 5 years	211	14.9
	5–10 years	181	12.8
	11–20 years	345	24.4
	21–30 years	211	14.9
	More than 30 years	467	33.0
Specialization status	Completed specialization	580	41.0
	Completed subspecialization	191	13.5
	No completed specialization/subspecialization	644	45.5
Level of healthcare delivery	Primary healthcare	473	33.4
	Secondary healthcare	614	43.4
	Tertiary healthcare	328	23.2
Ownership of healthcare organization	Public	1100	77.7
	Mixed ownership	299	21.1
	Private	16	1.1
Professional role	Medical doctors with specialization	474	33.5
	Nurses/medical technicians/dental nurses/midwives/OR nurses	379	26.8
	Medical doctors with subspecialization	188	13.3
	Medical doctors without specialization	70	4.9
	Other healthcare-related professions	304	21.5
Organizational position	Non-managerial	1099	77.7
	Managerial	316	22.3
ESG implementation status	Fully implemented	335	23.7
	Partially implemented	577	40.8
	Implementation underway	117	8.3
	Not implemented	311	22.0
	Unfamiliar with ESG status	74	5.2

**Note.** Percentages are based on the full sample (*N* = 1415). For categories where exact frequencies were not explicitly reported in the text, *n* values were calculated from the reported percentages and rounded to the nearest whole number; therefore, minor discrepancies may occur due to rounding.

**Table 2:** Pattern Matrix of the Four-Factor Measurement Structure.

	Factor			
	Environmental Sustainability	Social Sustainability	Environmental Financial Literacy	Governance/ESG Reporting
Employee Monitoring	−0.049	0.604	−0.023	−0.051
Social Decisions	0.005	0.703	−0.002	0.020
Social Performance	−0.012	0.618	−0.030	−0.033
Social Transparency	−0.002	0.674	0.027	−0.016
Social Monitoring	0.019	0.618	0.020	−0.014
Stakeholder Relations	0.052	0.680	0.002	0.025
Social Implementation	0.012	0.700	−0.013	−0.027
Employee Safety	−0.004	0.722	−0.011	−0.001
Professional Development	−0.001	0.597	0.067	0.102
Equal Treatment	−0.012	0.582	0.045	0.023
Workplace Equality	0.017	0.771	0.022	0.026
Employee Satisfaction	0.016	0.728	0.001	0.052
Patient Safety	0.046	0.655	−0.031	0.002
Patient Satisfaction	0.021	0.599	0.020	−0.007
Patient Rights	0.020	0.717	−0.022	−0.019
Environmental Costs	−0.025	0.050	0.691	−0.021
Green Investments	−0.020	−0.039	0.611	−0.038
Resource Efficiency	−0.001	−0.015	0.620	−0.074
Environmental Reporting	0.055	0.029	0.613	0.028
Sustainable Investment	0.012	0.018	0.702	0.000
Environmental Compliance	−0.007	0.008	0.742	0.007
Energy Reduction	0.670	0.028	0.024	0.007
Energy Efficiency	0.612	0.018	0.013	0.003
Resource Monitoring	0.664	−0.008	−0.024	−0.028
Impact Reduction	0.712	0.015	0.006	0.048
Renewable Energy	0.673	−0.014	−0.012	−0.045
Waste Disposal	0.592	−0.044	0.040	0.005
Hazardous Waste	0.683	0.032	−0.042	0.006
Environmental Training	0.629	0.009	0.042	0.017
Green Procurement	0.647	−0.010	−0.009	−0.022
Waste Monitoring	0.673	0.030	0.004	0.028
Digital Training	0.654	−0.034	−0.023	−0.072
Resource Reduction	0.743	−0.023	0.043	−0.021
Consumption Monitoring	0.775	0.012	0.073	0.060
Financial Transparency	0.034	0.035	−0.017	−0.548
Strategic Decisions	0.035	0.029	0.026	−0.512
Risk Management	0.002	0.049	0.095	−0.645
Internal Controls	0.032	0.028	0.025	−0.693
Ethical Accountability	−0.039	−0.008	0.026	−0.780
Information Quality	0.013	−0.034	0.054	−0.615

**Table 3:** Construct Reliability and Convergent Validity of the Measurement Model.

Construct	Cronbach’s α	ρA	ρC (CR)	AVE
F1—Environmental Sustainability	0.919	0.922	0.919	0.450
F2—Social Sustainability	0.923	0.926	0.923	0.447
F3—Environmental Financial Literacy	0.836	0.840	0.836	0.461
F4—Governance/ESG Reporting	0.830	0.839	0.832	0.456

**Table 4:** Discriminant Validity: Fornell–Larcker Criterion and HTMT Ratios.

Construct	F1	F2	F3	F4
F1—Environmental Sustainability	0.671	0.339	0.460	0.628
F2—Social Sustainability	0.660	0.669	0.436	0.555
F3—Environmental Financial Literacy	0.460	0.440	0.679	0.659
F4—Governance/ESG Reporting	0.620	0.550	0.340	0.675

Note. Diagonal values represent the square roots of average variance extracted (AVE). Values below the diagonal represent inter-construct correlations (Fornell–Larcker criterion), while values above the diagonal represent heterotrait–monotrait ratios (HTMT).

**Table 5:** Structural Model Results and Explained Variance.

Hypothesis	Structural Path	β	SE	C.R.	*p*-Value	R^2^	Result
H1	EFL → Governance/ESG Reporting	0.658	0.034	17.130	<0.001	0.433	Supported
H2	Governance/ESG Reporting → Environmental Sustainability	0.561	0.049	12.638	<0.001	0.391	Supported
H3	Governance/ESG Reporting → Social Sustainability	0.469	0.050	10.928	<0.001	0.315	Supported

Note. EFL = Environmental Financial Literacy; β = standardized path coefficient; SE = standard error; C.R. = critical ratio; R^2^ = proportion of variance explained in the corresponding endogenous construct. R^2^ values refer to the full structural model, including the direct paths from EFL to Environmental Sustainability and Social Sustainability, rather than to individual paths.

**Table 6:** Mediation Effects (Standardized).

Hypothesis	Indirect Path	Direct Effect (β)	Direct Effect *p*	Indirect Effect (β)	95% BC CI	Mediation Type
H4	Environmental Financial Literacy (EFL) → Governance/ESG Reporting → Environmental Sustainability	0.092	0.014	0.369	[0.330, 0.415]	Partial mediation
H5	Environmental Financial Literacy (EFL) → Governance/ESG Reporting → Social Sustainability	0.127	0.001	0.309	[0.266, 0.353]	Partial mediation

## Data Availability

The original contributions presented in this study are included in the article. Further inquiries can be directed to the corresponding author(s).

## Appendix A

**Table A1:** Measurement Items, Operational Definitions, Supporting References, and Item Retention Following Exploratory Factor Analysis.

Code	Item Title	Operational Definition	Supporting References	Final Factor	Status
V1	Social Responsibility	Consideration of broader social responsibilities in organizational practices.	[50,78]	—	Not retained
V2	Employee Monitoring	Monitoring of employee-related conditions and organizational practices.	[78,73]	Social Sustainability	Retained
V3	Stakeholder Communication	Communication with relevant internal and external stakeholders.	[50,81]	—	Not retained
V4	Social Decisions	Incorporation of social considerations into organizational decision-making.	[50,78]	Social Sustainability	Retained
V5	Social Performance	Assessment of organizational performance concerning social responsibilities.	[78,73]	Social Sustainability	Retained
V6	Social Transparency	Transparency regarding organizational social practices and responsibilities.	[15,78]	Social Sustainability	Retained
V7	Social Monitoring	Systematic monitoring of social sustainability practices and outcomes.	[78,81]	Social Sustainability	Retained
V8	Stakeholder Relations	Management of relationships with organizational stakeholder groups.	[50,81]	Social Sustainability	Retained
V9	Social Implementation	Implementation of socially responsible organizational practices.	[78,73]	Social Sustainability	Retained
V10	Employee Safety	Organizational measures supporting employee health and occupational safety.	[50,78]	Social Sustainability	Retained
V11	Professional Development	Organizational support for employee training and professional development.	[50,78]	Social Sustainability	Retained
V12	Equal Treatment	Fair and equitable treatment of employees within the organization.	[50,78]	Social Sustainability	Retained
V13	Workplace Equality	Promotion of equality and non-discrimination in the workplace.	[50,78]	Social Sustainability	Retained
V14	Employee Satisfaction	Organizational attention to employee satisfaction and well-being.	[50,78]	Social Sustainability	Retained
V15	Patient Safety	Organizational measures aimed at protecting patient safety.	[78,81]	Social Sustainability	Retained
V16	Patient Satisfaction	Consideration and monitoring of patient satisfaction with healthcare services.	[78,81]	Social Sustainability	Retained
V17	Patient Rights	Protection and consideration of patients’ rights in organizational practices.	[50,81]	Social Sustainability	Retained
V18	Equal Access	Organizational commitment to equitable access to healthcare services.	[50,93,94]	—	Not retained
V19	Balanced Decisions	Consideration of financial, environmental, and social priorities in organizational decisions.	[40,50]	—	Not retained
V20	Green Decisions	Incorporation of environmental considerations into organizational decision-making.	[54,59]	—	Not retained
V21	Environmental Costs	Capability to evaluate environmental costs and their financial implications.	[54,59]	Environmental Financial Literacy	Retained
V22	Green Investments	Capability to assess the financial implications of environmentally oriented investments.	[54,59]	Environmental Financial Literacy	Retained
V23	Resource Efficiency	Capability to evaluate resource efficiency from financial and environmental perspectives.	[19,54,59]	Environmental Financial Literacy	Retained
V24	Environmental Reporting	Capability to interpret environmental reporting information for financially informed decisions.	[23,24,68]	Environmental Financial Literacy	Retained
V25	Sustainable Investment	Capability to evaluate investments incorporating sustainability-related financial considerations.	[25,54]	Environmental Financial Literacy	Retained
V26	Environmental Compliance	Capability to assess environmental compliance requirements and associated financial implications.	[54,59]	Environmental Financial Literacy	Retained
V27	Energy Reduction	Implementation of organizational measures aimed at reducing energy consumption.	[6,7,71]	Environmental Sustainability	Retained
V28	Energy Efficiency	Adoption of energy-efficient technologies and operational practices.	[7,71]	Environmental Sustainability	Retained
V29	Resource Monitoring	Systematic monitoring of resource consumption within the organization.	[6,61]	Environmental Sustainability	Retained
V30	Impact Reduction	Organizational measures aimed at reducing environmental impacts.	[1,6]	Environmental Sustainability	Retained
V31	Renewable Energy	Organizational adoption or promotion of renewable energy sources.	[1,7]	Environmental Sustainability	Retained
V32	Waste Disposal	Implementation of environmentally responsible waste disposal practices.	[1,6]	Environmental Sustainability	Retained
V33	Hazardous Waste	Management and disposal of hazardous healthcare waste according to environmental requirements.	[1,60]	Environmental Sustainability	Retained
V34	Waste Reduction	Measures aimed at minimizing organizational waste generation.	[1,6]	—	Not retained
V35	Environmental Training	Provision of environmental training and sustainability awareness activities.	[1,60]	Environmental Sustainability	Retained
V36	Green Procurement	Integration of environmental criteria into organizational procurement practices.	[4,6]	Environmental Sustainability	Retained
V37	Waste Monitoring	Systematic monitoring of waste generation and management.	[1,6]	—	Not retained
V38	Digital Training	Training employees to use digital technologies in organizational activities.	[16,17]	—	Not retained
V39	Waste Control	Application of organizational controls for waste management practices.	[1,6]	Environmental Sustainability	Retained
V40	Digital Reduction	Use of digital technologies to support reductions in environmental impacts or resource consumption.	[16,57]	Environmental Sustainability	Retained
V41	Lifecycle Management	Consideration of environmental impacts throughout the life cycle of organizational resources or products.	[4,57]	Environmental Sustainability	Retained
V42	Resource Reduction	Implementation of practices aimed at reducing organizational resource consumption.	[6,61]	Environmental Sustainability	Retained
V43	Consumption Monitoring	Monitoring of organizational consumption patterns to support environmental management.	[6,7]	Environmental Sustainability	Retained
V44	Digital Procurement	Application of digital technologies to organizational procurement processes.	[16,57]	—	Not retained
V45	Financial Transparency	Transparency and accessibility of organizational financial information and reporting.	[15,68]	Governance/ESG Reporting	Retained
V46	Management Quality	Quality of organizational management, oversight, and governance processes.	[64,67]	—	Not retained
V47	Strategic Decisions	Integration of financial and sustainability considerations into organizational strategic decisions.	[40,68]	Governance/ESG Reporting	Retained
V48	Risk Management	Identification, assessment, and management of organizational and sustainability-related risks.	[64,67]	Governance/ESG Reporting	Retained
V49	Internal Controls	Implementation of internal control mechanisms supporting organizational accountability and oversight.	[66,68]	Governance/ESG Reporting	Retained
V50	Ethical Accountability	Application of ethical responsibility and accountability principles in organizational governance.	[65,78]	Governance/ESG Reporting	Retained
V51	Information Quality	Reliability, accuracy, and usefulness of information supporting governance and ESG reporting.	[15,77]	Governance/ESG Reporting	Retained

**Note.** The initial questionnaire comprised 51 measurement items, of which 41 were retained following exploratory factor analysis. The retained items formed four factors: Environmental Sustainability (14 items), Social Sustainability (15 items), Environmental Financial Literacy (6 items), and Governance/ESG Reporting (6 items). Item retention was based on factor loadings, cross-loadings, and theoretical interpretability. Operational definitions describe the intended conceptual content of individual items, while supporting references indicate literature informing their development rather than sources of verbatim item adoption. The final factor structure was determined empirically.
